# From lipid profiles to disease onset: Triglycerides as predictive biomarkers in endometriosis

**DOI:** 10.1097/MD.0000000000047910

**Published:** 2026-03-06

**Authors:** Qi Wu, Qi Zhang, Yifu Tie, Guangyu Cheng

**Affiliations:** a Graduate School, Heilongjiang University of Chinese Medicine, Harbin, China; b Center for Preventive Treatment of Disease, First Affiliated Hospital, Heilongjiang University of Chinese Medicine, Harbin, China; c Traditional Chinese Medicine Translational Medicine Research Center, First Affiliated Hospital, Heilongjiang University of Chinese Medicine, Harbin, China.

**Keywords:** causal inference, cross-sectional study, dyslipidemia, endometriosis, triglycerides

## Abstract

Dyslipidaemia is related to endometriosis, but it is not known which lipid component is most relevant, and whether the observed correlation reflects the causal relationship. The relationship between triglycerides (TG) and endometriosis is studied through population research, and Mendelian randomization (MR) is used to evaluate the genetic evidence consistent with the causal relationship. We analyzed 2345 women from National Health and Nutrition Examination Survey 1999 to 2006 to assess lipid profiles and self-reported clinician-diagnosed endometriosis. Survey-weighted multivariable logistic regression was used with progressive covariate adjustment. Lipid-lowering medication use was additionally considered to address treatment-related confounding. For causal inference, 2-sample MR was conducted using genome-wide significant instruments for TG from large genome-wide association study resources and endometriosis summary statistics from FinnGen. Sensitivity analyses included assessments of heterogeneity and horizontal pleiotropy, as well as leave-one-out analyses. In the cross-sectional analysis, higher TG levels were associated with higher odds of endometriosis after multivariable adjustment. Compared with the lowest TG quartile, the highest quartile showed increased odds (odds ratio = 1.71, 95% confidence interval: 1.01–2.89). In MR analyses, genetically predicted TG was associated with endometriosis with consistent direction across methods; the inverse variance weighted estimate was odds ratio = 1.25 (95% confidence interval: 1.11–1.40). No statistically significant associations were observed for total cholesterol, low-density lipoprotein, or high-density lipoprotein. Subgroup analyses did not suggest clear effect heterogeneity by age, body mass index, or major metabolic comorbidities. In National Health and Nutrition Examination Survey 1999 to 2006, elevated TG was associated with higher odds of endometriosis. Two-sample MR provided genetic evidence consistent with a causal contribution of lifelong higher TG to endometriosis risk. These findings warrant confirmation in prospective cohorts and interventional studies before translation into clinical prevention strategies.

## 1. Introduction

Endometriosis is an estrogen-dependent inflammatory disease characterized by the ectopic transplantation of functional endometrial matrix and glands, which brings major clinical problems to the health management of women around the world.^[1]^ Epidemiological surveys estimate that during the fertility window period, the prevalence rate is 1 in 10 women,^[2]^ and the main clinical manifestations are chronic pelvic pain, disabilities related to dysuria, and reproductive disfunction.^[3]^ Although the disease gradually leads to a decline in the quality of life and causes multi-organ complications, the pathophysiological mechanism and molecular driving factors in its development process cannot be clearly described, which has caused certain difficulties for targeted treatment innovation. Previous studies have shown that endocrine disorders, immune system abnormalities, genetic factors, and environmental influences will all promote its occurrence.^[4]^ Investigating the role of blood lipids in endometriosis can understand its cause and improve the methods of prevention and treatment.

Blood lipids, that is, total cholesterol (TCHO), triglycerides (TG) and lipoprotein,^[5]^ etc, play a very key role in many physiological processes of the human body, such as hormone synthesis and inflammatory reaction.^[6]^ In gynecology, hyperlipidemia is considered to be related to the occurrence and development of many gynaecological diseases such as endometriosis.^[7]^ However, the relationship between blood lipids and endometriosis has not been studied.

This paper adopts a cross-sectional design and uses the National Health and Nutrition Examination Survey (NHANES) database to study the relationship between circulating lipid spectrum and endometriosis. NHANES is an important epidemiological data that can reflect differences in the prevalence of various diseases in the US population, population distribution, and changeability determinants of risk.^[8]^ In order to further determine the scope of the investigation, we conducted a weighted observation analysis in NHANES, and used the Mendelian randomization (MR) method^[9]^ to test whether the association of TG-endometriosis is causal. Use comprehensive methods to strengthen causal inference and provide possible information for future mechanism and intervention research.

## 2. Materials and methods

### 2.1. A cross-sectional study based on NHANES

#### 2.1.1. Study population and design

**Figure 1.**
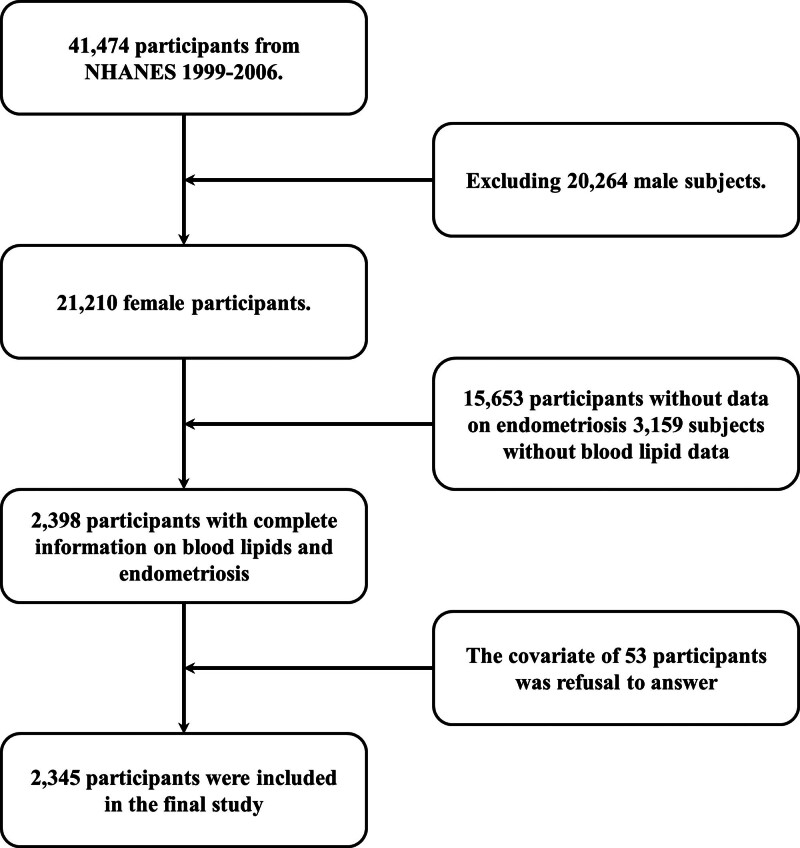
A flowchart for the inclusion and exclusion of cross-sectional studies. NHANES = National Health and Nutrition Examination Survey.

The NHANES, administered by the National Center for Health Statistics, is a nationwide initiative designed to monitor health conditions and dietary intake patterns across the US population. Its protocol incorporates multitiered ethical review mechanisms and employs a tripartite data collection framework comprising systematic interviews, standardized physical evaluations, and biospecimen analyses. Our study specifically assessed the relationship between lipid profiles and endometriosis in women. From 1999 to 2020, blood lipid data were recorded in the NHANES database, but endometriosis data were only available from 1999 to 2006. Thus, our cross-sectional study primarily used data from these 4 cycles (n = 41,474). We excluded male subjects (n = 20,264), participants with missing data on endometriosis diagnosis (n = 15,653) or lipid data (n = 3159), and those with uncertain or refused responses to covariates (n = 53), resulting in 2345 eligible subjects (Fig. 1).

#### 2.1.2. Diagnosis of endometriosis

Endometriosis diagnosis was based on a reproductive health questionnaire for women aged 20 to 54 years conducted between 1999 and 2006, including the question: “Has a doctor or other health professional ever told you that you have endometriosis?” Participants who answered affirmatively were classified as having endometriosis. Although self-report is not equivalent to surgical confirmation, large population studies using similar items have shown acceptable agreement with medical records, indicating that misclassification is likely non-differential and would bias our risk estimates toward the null rather than inflate them.

#### 2.1.3. Lipid profile measurements

Lipid indicators included TCHO, TG, low-density lipoprotein (LDL), and high-density lipoprotein (HDL), collected from laboratory tests conducted from 1999 to 2006 in NHANES participants. Blood samples were collected, handled, and transported for analysis at the Lipoprotein Analytical Laboratory of Johns Hopkins University. TCHO levels were enzymatically determined using Roche Hitachi analyzers (models 717 and 912). TG were quantified enzymatically through coupled biochemical reactions. HDL-C quantification adhered to standardized clinical laboratory protocols, employing either heparin-manganese precipitation separation techniques or chemiluminescent immunoassays. LDL-C estimation was derived from the Friedewald equation: LDL-C = TCHO − HDL-C − (TG/2.2) when values are expressed in mmol/L, with strict application limited to specimens demonstrating triglyceride concentrations <4.52 mmol/L.

#### 2.1.4. Other covariates

Covariates were selected based on baseline characteristics and dietary intake. Baseline characteristics included age, race, education level, marital status, pregnancy status at examination, age at menarche, body mass index (BMI), diabetes status, smoking status, drinking status, and poverty-to-income ratio (PIR). Age was categorized as <40 years or ≥40 years. Participants were classified racially into categories of Mexican American, other Hispanic, non-Hispanic White, non-Hispanic Black, or other races. Educational attainment was grouped as follows: below 9th grade, 9 to 11th grade, high school graduate/general education development equivalent, some college or associate degree, and college graduate or higher. Marital status was recorded as married or unmarried. Pregnancy status was categorized as pregnant, not pregnant, or uncertain. Age at menarche was categorized as <11 years, 11 to 13 years, and ≥14 years. BMI was calculated as weight (kg)/height^2^ (m^2^) and categorized into 3 groups: <25, 25 to 30, and >30. Diabetes was classified as yes/no/borderline based on the question “Has a doctor ever told you that you have diabetes?” Smoking status was classified as nonsmokers and smokers, with nonsmokers defined as those who had never smoked 100 cigarettes in their lifetime and smokers as those who had smoked 100 cigarettes. Drinking status was categorized into drinkers, defined as those who had at least 12 drinks/yr, and nondrinkers, defined as those who did not meet this criterion. PIR was used to evaluate household income. Dietary intake included total fat intake and TCHO intake from dietary questionnaires. To address potential confounding by treatment, we additionally accounted for lipid-lowering medication use based on the NHANES prescription medication questionnaire. Participants were classified as “users” if they reported current use of lipid-lowering agents (e.g., statins, fibrates, niacin, bile acid sequestrants, or other TG-lowering medications) and “nonusers” otherwise. We summarized lipid-lowering medication use overall and across TG quartiles, and conducted a sensitivity analysis excluding medication users to evaluate robustness.

#### 2.1.5. Statistical analysis for cross-sectional study

All statistical analyses combine the corresponding sampling weights and variance estimation methods, reflecting the complex sampling design of NHANES, taking into account oversampling, nonresponsive deviation and stratification. NHANES has set up a special weighted scheme to regulate oversampling groups, incomplete survey responses, and demographic stratification in line with the Census Bureau’s estimates. Baseline demographics are stratified according to the symptoms of endometriosis. Classified variables are analyzed by the weighted frequency distribution represented by proportion, and continuous variables are analyzed by the quantified weighted average (standard error). For intergroup comparison, the method of hierarchical analysis is used to analyze the weighted linear regression model with complex sampling weights, and the classification variables are processed by the investigation weighted card square test adjusted by the sampling design effect. In order to ensure the validity of statistics and prevent deviations caused by missing data, the multiple extrapolation algorithm of chain equations is used to calculate the missing values (PIR, BMI, total fat, TCHO intake). Using multivariate logical regression, research is carried out on the basis of investigating the relationship between lipid markers and endometriosis in 3 models, that is, model 1 (rough, unadjusted), model 2 (adjusted according to age, race, education, PIR), model 3 (comprehensively adjustment). Subgroup analyses were performed by stratifying according to factors such as age, race, education, marital status, pregnancy status at examination, age at menarche, BMI, diabetes, smoking, alcohol use, PIR, total fat, and TCHO intake. Interaction terms were also tested to identify potential heterogeneity between subgroups. Survey-weighted analyses were conducted in R (v4.2.1). EmpowerStats (v2.0) was used as an auxiliary platform for weighted modeling and result verification.

### 2.2. Research on causality based on MR

#### 2.2.1. Study design

**Figure 2.**
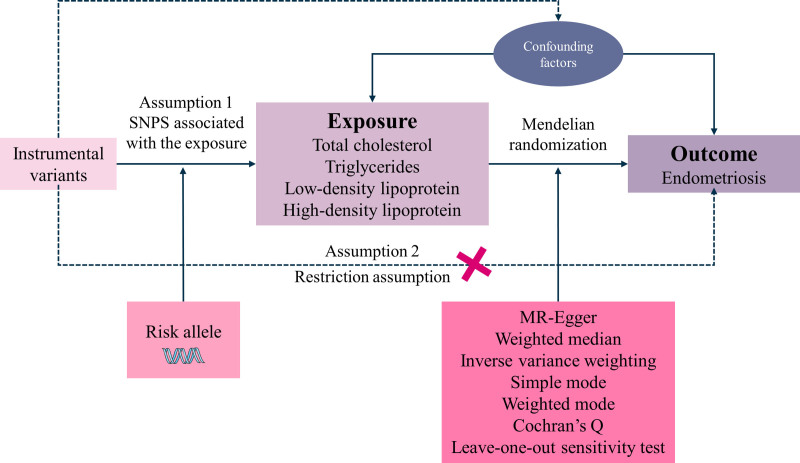
Design of the MR study. MR = Mendelian randomization.

This analysis adopted a 2-sample, MR design. Both exposure and outcome populations were restricted to individuals of European ancestry to minimize confounding caused by population stratification. Instrumental variables (IVs) in MR satisfy 3 basic assumptions (Fig. 2).

#### 2.2.2. Selection of instrument variables

For IV selection, we set a genome-wide significance threshold at *P* < 5E−08. To ensure independence among selected single nucleotide polymorphism (SNPs) and eliminate potential pleiotropy, linkage disequilibrium was restricted to *r*^2^ < 0.001 within a 10,000 kb genomic range. The primary data sources were the UK Biobank, FinnGen Biobank, and Integrative Epidemiology Unit open genome-wide association study (GWAS) database (Table 1). Exposures included ebi-a-GCST90092985 (TCHO, variants = 1,15,90,399), ebi-a-GCST90014014 (TG, variants = 1,07,83,708), ebi-a-GCST90014007 (HDL, variants = 1,07,83,660), and ieu-b-4846 (LDL, variants = 78,92,997). The outcome was finn-b-N14_ENDOMETRIOSIS (endometriosis, variants = 1,63,77,306). After identifying GWAS data for lipid levels and endometriosis, genome-wide significant SNPs associated with cholesterol, TG, LDL, and HDL were selected from the corresponding exposure GWAS datasets, and the associations of these instruments with endometriosis were extracted from the FinnGen outcome summary statistics. To maintain consistent directionality of SNP effects across datasets, allele orientations were harmonized using the data.table package. Moreover, Mendelian randomization pleiotropy residual sum and outlier was applied to identify and exclude outlier SNPs exhibiting horizontal pleiotropy, as well as SNPs directly associated with the outcome (*P* < 5E−08). SNP strength was evaluated using the *F*-statistic (*F* = β^2^_exposure/SE^2^_exposure), and SNPs with an *F*-statistic below 10 were excluded to prevent biases from weak IVs. After harmonization and quality control, the numbers of instruments included in each exposure were: TG n = 281 (Table S1, Supplemental Digital Content, https://links.lww.com/MD/R486), LDL n = 55 (Table S2, Supplemental Digital Content, https://links.lww.com/MD/R486), HDL n = 48 (Table S3, Supplemental Digital Content, https://links.lww.com/MD/R486), and TCHO n = 326 (Table S4, Supplemental Digital Content, https://links.lww.com/MD/R486). Instrument strength was consistently adequate: TG *F*_min = 30.11, *F*_median = 49.67, *F*_mean = 137.50; LDL *F*_min = 31.03, *F*_median = 56.99, *F*_mean = 99.71; HDL *F*_min = 30.53, *F*_median = 60.12, *F*_mean = 145.41; TCHO *F*_min = 30.06, *F*_median = 54.29, *F*_mean = 141.85 (all *F* > 10). These statistics indicate no evidence of weak-instrument bias across exposures.

**Table 1:** Summary of genome-wide association study data.

	Phenotype	Data source	GWAS id	Sample size	Number of SNPs	Race	Year	PMID
Exposure	TCHO	Richardson TG	ebi-a-GCST90092985	1,15,082	1,15,90,399	European	2022	35213538
	TG	Mbatchou J	ebi-a-GCST90014014	3,89,562	1,07,83,708	European	2021	34017140
	LDL	Mbatchou J	ebi-a-GCST90014007	3,57,810	1,07,83,660	European	2021	34017140
	HDL	Howe LJ	ieu-b-4846	70,814	78,92,997	European	2022	NA
Outcome	Endometriosis	NA	finn-b-N14_ENDOMETRIOSIS	77,257	1,63,77,306	European	2021	NA

GWAS = genome-wide association study, HDL = high-density lipoprotein, LDL = low-density lipoprotein, PMID = pubmed Identifier, SNP = single nucleotide polymorphism, TCHO = total cholesterol, TG = triglycerides.

#### 2.2.3. MR statistical analysis

Five MR methods were employed to analyze the data, including inverse variance weighted (IVW), MR-Egger, simple mode, weighted mode, and weighted median (WMD).^[10]^ We conducted magnetic resonance analysis of cholesterol, TG, LDL and HDL of endometriosis, and *P* < .05 was significant. In order to test whether more IVs will cause heterogeneity to cause and effect, Cochran’s *Q* test was done on the results of cause and effect. In addition, the IV difference in the size of a single effect can cause polydirectional heterogeneity, which is tested by MR-Egger regression. In MR-Egger regression, the intercept of *P* > .05 is close to 0, indicating that there is no polyposis. In addition, we also use Mendelian randomization pleiotropy residual sum and outlier global test to detect horizontal polysotropority and potential out-of-group value instruments, and interpret the consistency of IVW/WMD/mode-based estimators as supportive, thus indicating that the results are not caused by the single variable effect. Use the PhenoScanner database to test the relationship between causal SNP and potential confounding factors. Use the leaf-one-out method for sensitivity analysis to evaluate the stability of the IVW estimate and find out all individual SNPs that may affect the overall correlation. Generate a funnel diagram to intuitively evaluate potential biases. All statistical analyses are used on R (version 4.2.3; Vienna, Austria), and the TwoSample MR package (version 0.5.6; Vienna, Austria) is used.

## 3. Results

### 3.1. Cross-sectional study

#### 3.1.1. Characteristics of study participants

After excluding the subjects with endometriosis and lipid data loss, a total of 2255 women participated in this study. The prevalence of endometriosis is about 7.87%, with a total of 171 cases. Compared with women who have not been diagnosed with endometriosis, women over the age of 40 and married women have a higher incidence rate. Similarly, the number of women in the menstrual period between the ages of 11 and 13 is also higher. There are large differences between endometriosis and non-endometriosis groups in terms of age, race, education, marital status, pregnancy, smoking, drinking, and PIR (Table 2).

**Table 2:** Weighted comparison in basic characteristics.

Variables	Total (n = 2345)	No endometriosis (n = 2174)	Endometriosis (n = 171)	Statistics	*P*
Age, n (%)				0.35 (0.20, 0.51)	<.001
<40	1448 (61.75%)	1370 (63.02%)	78 (45.61%)		
≥40	897 (38.25%)	804 (36.98%)	93 (54.39%)		
Race, n (%)				0.61 (0.46, 0.77)	<.001
Mexican American	556 (23.71%)	546 (25.11%)	10 (5.85%)		
Other Hispanic	105 (4.48%)	100 (4.60%)	5 (2.92%)		
Non-Hispanic White	1093 (46.61%)	977 (44.94%)	116 (67.84%)		
Non-Hispanic Black	487 (20.77%)	453 (20.84%)	34 (19.88%)		
Other race	104 (4.43%)	98 (4.51%)	6 (3.51%)		
Education level, n (%)				0.42 (0.26, 0.57)	.001
<9th grade	208 (8.87%)	206 (9.48%)	2 (1.17%)		
9–11th grade	357 (15.22%)	338 (15.55%)	19 (11.11%)		
High school graduate/GED or equivalent	500 (21.32%)	456 (20.98%)	44 (25.73%)		
Some college or AA degree	769 (32.79%)	707 (32.52%)	62 (36.26%)		
College graduate or above	511 (21.79%)	467 (21.48%)	44 (25.73%)		
Marital status, n (%)				0.23 (0.07, 0.39)	.006
Married	1572 (67.04%)	1441 (66.28%)	131 (76.61%)		
Never married	773 (32.96%)	733 (33.72%)	40 (23.39%)		
Pregnancy status, n (%)				0.28 (0.13, 0.44)	.006
Yes	449 (19.15%)	432 (19.87%)	17 (9.94%)		
No	1846 (78.72%)	1697 (78.06%)	149 (87.13%)		
Cannot ascertain	50 (2.13%)	45 (2.07%)	5 (2.92%)		
Age at menarche, n (%)				0.18 (0.02, 0.33)	.089
<11	206 (8.78%)	186 (8.56%)	20 (11.70%)		
11–13	1557 (66.40%)	1438 (66.15%)	119 (69.59%)		
≥14	582 (24.82%)	550 (25.30%)	32 (18.71%)		
BMI, n (%)				0.10 (−0.06, 0.25)	.467
<25	806 (34.37%)	752 (34.59%)	54 (31.58%)		
25–30	681 (29.04%)	634 (29.16%)	47 (27.49%)		
≥30	858 (36.59%)	788 (36.25%)	70 (40.94%)		
Diabetes, n (%)				0.02 (−0.13, 0.18)	.964
Yes	90 (3.84%)	84 (3.86%)	6 (3.51%)		
No	2243 (95.65%)	2079 (95.63%)	164 (95.91%)		
Borderline	12 (0.51%)	11 (0.51%)	1 (0.58%)		
Smoking, n (%)				0.30 (0.14, 0.46)	<.001
Yes	886 (37.78%)	798 (36.71%)	88 (51.46%)		
No	1459 (62.22%)	1376 (63.29%)	83 (48.54%)		
Drinking, n (%)				0.20 (0.04, 0.36)	.015
Yes	1426 (60.81%)	1307 (60.12%)	119 (69.59%)		
No	919 (39.19%)	867 (39.88%)	52 (30.41%)		
Lipid-lowering medication use, n (%)				0.11 (−0.04, 0.25)	.1800
Yes	110 (4.7%)	98 (4.5%)	12 (7.0%)		
No	2235 (95.3%)	2076 (95.5%)	159 (93.0%)		
Family PIR, mean (SE)	2.69 (1.60)	2.66 (1.60)	3.06 (1.64)	0.24 (0.09, 0.40)	.002
Dietary fat intake, mean (SE)	75.65 (39.07)	75.66 (39.24)	75.53 (37.07)	0.00 (−0.15, 0.16)	.966
Dietary cholesterol intake, mean (SE)	266.07 (209.41)	267.92 (210.83)	242.54 (189.53)	0.13 (−0.03, 0.28)	.127
TCHO, mmol/L, mean (SE)	5.14 (1.11)	5.12 (1.11)	5.26 (1.09)	0.13 (−0.03, 0.28)	.115
TG, mmol/L, mean (SE)	1.42 (0.81)	1.42 (0.80)	1.50 (0.82)	0.10 (−0.06, 0.25)	.221
LDL, mmol/L, mean (SE)	3.00 (0.92)	2.97 (0.91)	3.07 (0.96)	0.11 (−0.04, 0.27)	.144
HDL, mmol/L, mean (SE)	1.51 (0.42)	1.51 (0.42)	1.50 (0.44)	0.01 (−0.15, 0.16)	.911

Weighted mean (SE) for continuous variables.

*P* value was calculated by weighted linear regression model.

% for categorical variables: *P* value was calculated by weighted chi-square test.

AA = associate of arts, BMI = body mass index, GED = general education development, HDL = high-density lipoprotein, LDL = low-density lipoprotein, PIR = poverty-to-income ratio, TCHO = total cholesterol, TG = triglycerides.

#### 3.1.2. Association between lipid levels and endometriosis risk

In survey-weighted logistic regression, TG was positively associated with endometriosis (Table 3). After adjustment for age, race, education level, and PIR, the association remained statistically significant (odds ratio [OR] = 1.25, 95% confidence interval [CI]: 1.03–1.52, *P* = .027). With further adjustment for additional covariates, the estimate was similar (OR = 1.31, 95% CI: 1.04–1.64, *P* = .019), indicating that each 1 mmol/L increase in TG was associated with 31% higher odds of endometriosis. A subset of participants in the highest TG quartile (Q4) reported lipid-lowering medication use. Overall, 110 participants (4.7%) reported lipid-lowering medication use; the proportion was 4.5% in women without endometriosis and 7.0% in women with endometriosis (Table 2). Additional adjustment for lipid-lowering medication use did not materially alter the association estimates. Conversely, no significant associations were found between TCHO, LDL, or HDL levels and endometriosis in any model. In quartile analyses, TG showed a threshold pattern: compared with Q1, Q4 was associated with higher odds of endometriosis in model 2 (OR = 1.64, 95% CI: 1.02–2.63, *P* = .040) and model 3 (OR = 1.71, 95% CI: 1.01–2.89, *P* = .046). However, no significant association was found between the lowest quartile of TG and endometriosis. These results suggest that higher TG levels, particularly in the highest quartile, are associated with higher odds of endometriosis in this cross-sectional sample. Participants in the highest TG quartile had 76% higher odds of endometriosis compared with those in the lowest quartile.

**Table 3:** Association between blood lipids and endometriosis.

	Model 1, OR (95% CI) *P*	Model 2, OR (95% CI) *P*	Model 3, OR (95% CI) *P*
Blood lipids			
TCHO	1.11 (0.97–1.28) .116	1.06 (0.92–1.23) .404	1.08 (0.93–1.27) .317
TG	1.12 (0.93–1.35) .221	1.25 (1.03–1.52) .027	1.31 (1.04–1.64) .019
LDL	1.13 (0.96–1.33) .144	1.03 (0.87–1.23) .713	1.03 (0.86–1.23) .761
HDL	0.98 (0.68–1.42) .911	0.91 (0.61–1.34) .623	1.01 (0.66–1.54) .972
Quartile of TG			
Q1	1 (Reference)	1 (Reference)	1 (Reference)
Q2	1.25 (0.79–1.98) .337	1.29 (0.81–2.06) .287	1.24 (0.77–2.00) .379
Q3	1.31 (0.83–2.07) .243	1.34 (0.84–2.15) .217	1.30 (0.80–2.12) .291
Q4	1.34 (0.85–2.11) .203	1.64 (1.02–2.63) .040	1.71 (1.01–2.89) .046

Model 1: no covariates were adjusted. Model 2: adjusted for age, race, education level, and PIR. Model 3: adjusted for age, race, education level, marital status, PIR, pregnancy status, age at menarche, BMI, diabetes, smoking, drinking, dietary fat intake, and dietary cholesterol intake, and lipid-lowering medication use.

BMI = body mass index, CI = confidence interval, HDL = high-density lipoprotein, LDL = low-density lipoprotein, OR = odds ratio, PIR = poverty-to-income ratio, TCHO = total cholesterol, TG = triglycerides.

#### 3.1.3. Subgroup analysis

**Figure 3.**
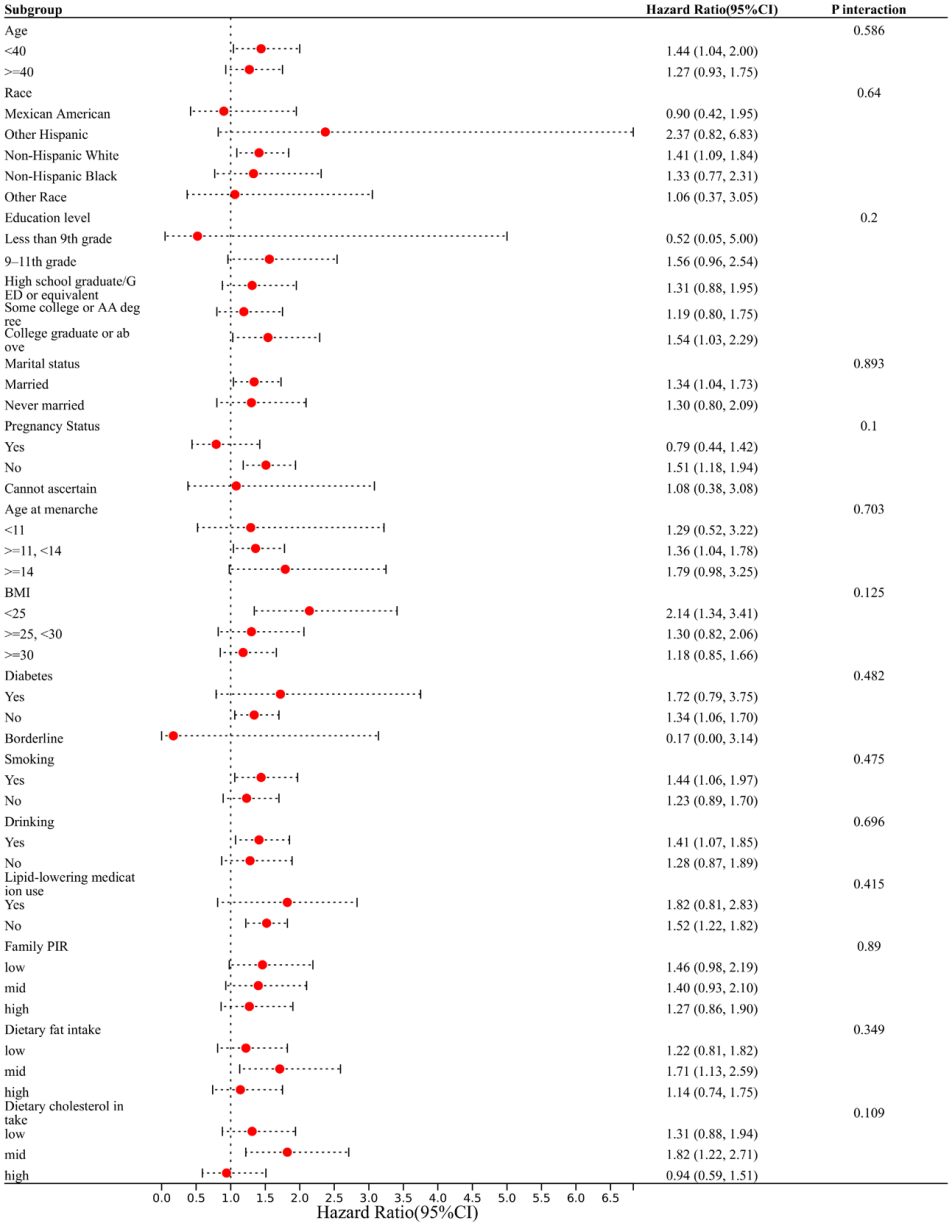
Subgroup analysis for the association between TG and endometriosis. AA = associate of arts, BMI = body mass index, CI = confidence interval, ED = endometriosis; PIR = poverty-to-income ratio, TG = triglycerides, UKB = UK Biobank.

Stratified regression modeling was implemented to assess the cross-dimensional robustness of the TG levels and endometriosis (Fig. 3). Interaction tests indicated no significant modification of the association by factors such as age, race, educational level, marital status, pregnancy status, age at menarche, BMI, diabetes, smoking, alcohol consumption, PIR, dietary fat intake, and dietary cholesterol intake. This suggests no evidence of effect heterogeneity across the prespecified subgroups.

### 3.2. MR analysis

We performed MR analysis for lipid levels and endometriosis. Each IV had an *F*-statistic > 10, indicating adequate instrument strength for the primary TG analysis (Table S1, Supplemental Digital Content, https://links.lww.com/MD/R486).

#### 3.2.1. MR analysis of TG and endometriosis

**Figure 4.**
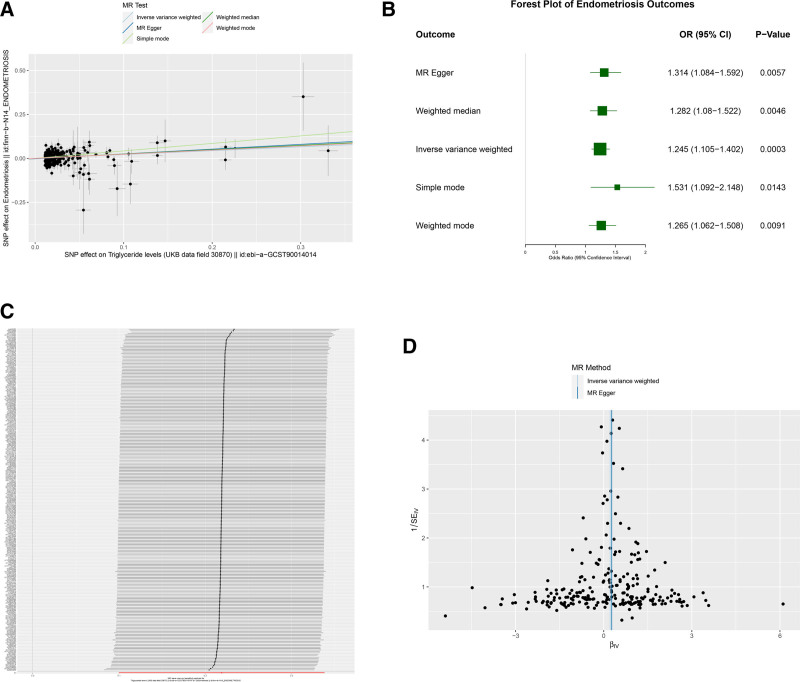
MR results for TG and endometriosis. (A) Scatter plot of SNP effects. (B) Forest plot of causal estimates across MR methods. (C) Leave-one-out analysis. (D) Funnel plot for assessing potential asymmetry. CI = confidence interval, ED = endometriosis; MR = Mendelian randomization, OR = odds ratio, SNP = single nucleotide polymorphism, TG = triglycerides, UKB = UK Biobank.

The MR framework incorporated 281 genome-wide significant TG-associated SNPs after rigorous quality control (Table S1, Supplemental Digital Content, https://links.lww.com/MD/R486). All 5 MR analysis methods were statistically significant (Table 4): MR-Egger (OR = 1.314, 95% CI: 1.084–1.592, *P* = .006); WMD (OR = 1.282, 95% CI: 1.080–1.522, *P* = .005); IVW (OR = 1.245, 95% CI: 1.105–1.402, *P* < .001); simple mode (OR = 1.531, 95% CI: 1.092–2.148, *P* = .014); and weighted mode (OR = 1.265, 95% CI: 1.062–1.508, *P* = .009). Importantly, effect estimates were directionally consistent across methods, indicating that the results were robust and consistent across complementary MR estimators and sensitivity analyses (Fig. 4A, B).

**Table 4:** The MR analysis results of TG and endometriosis.

Method	OR (95% CI)	*P*
MR-Egger	1.314 (1.084–1.592)	.006
Weighted median	1.282 (1.080–1.522)	.005
Inverse variance weighted	1.245 (1.105–1.402)	<.001
Simple mode	1.531 (1.092–2.148)	.014
Weighted mode	1.265 (1.062–1.508)	.009
heterogeneity test	MR Egger	<.05
	Inverse variance weighted	<.05
Pleiotropy test	Egger_intercept	−.002
	*P*	.481

CI = confidence interval, MR = Mendelian randomization, OR = odds ratio, TG = triglycerides.

Sensitivity analysis found that there is a certain heterogeneity (*Q* < 0.05) in evaluating the relationship between TG and endometriosis. However, the intercept of MR-Egger regression is 0, which is not statistically significant (intercept = −0.002, *P* = .481), indicating that there is no obvious horizontal polytropicity or deviation. Leave an analysis to show the stability of the IVW estimate when removing an SNP (Fig. 4C). The funnel diagram is asymmetrical, which makes the MR result stable (Fig. 4D). In summary, the influence direction of the MR method is the same, and the sensitivity analysis has found no strong evidence of directional polygonal sex, so it supports the causal effect of genetic prediction of TG on the risk of endometriosis.

#### 3.2.2. MR analysis of other lipid indicators and endometriosis

According to the magnetic resonance analysis of TCHO, LDL, and HDL, there are 55 SNPs of LDLs (Table S2, Supplemental Digital Content, https://links.lww.com/MD/R486), 48 SNPs of HDLs (Table S3, Supplemental Digital Content, https://links.lww.com/MD/R486), and 326 SNPs of TCHO (Table S4, Supplemental Digital Content, https://links.lww.com/MD/R486). None of the 5 MR methods showed a statistically significant correlation between these 3 fats and endometriosis (Table S5, Supplemental Digital Content, https://links.lww.com/MD/R486). The IVW analysis result of cholesterol and endometriosis is *P* = .667 > .05; the IVW analysis result of LDL and endometriosis is *P* = .483 > .05; the IVW analysis result of HDL and endometriosis is *P* = .331 > .05. The results of MR analysis did not give any evidence of the causal relationship between TCHO, LDL, or HDL and endometriosis.

## 4. Discussion

The main finding of the study is that there is a strong positive correlation between the increase in TG levels and the prevalence of endometriosis, especially in the highest quartile array of TG. In addition, this positive correlation is also supported by the MR test, which provides genetic evidence consistent with the causal relationship of high lifetime TG on the risk of endometriosis. From the relative width of 95% CI (1.01-2.89) of the Q4 odds, it can be seen that the incidence of endometriosis is low (7.9%), and complex survey weights can be used in quartile-based subgroups, which can reduce accuracy and produce a larger CI.

The results we found are roughly the same as the previous literature. The literature shows that lipid metabolism is associated with endometriosis, but this study strongly proves the causal link between TG levels and the disease. Previous reports have pointed out that the components of metabolic syndrome are associated with endometriosis, but there is no causal relationship.^[11]^ Our study added the necessary covariates to give genetic evidence consistent with the causal effect of higher TG on the risk of endometriosis by magnetic resonance analysis, reducing confusion and improving the robustness of the conclusion. Although magnetic resonance analysis shows that gene-driven hypertriglyceridemia plays a causal role in the pathogenesis of endometriosis, it cannot be accurately explained due to methodological limitations. MR estimates that it essentially reflects lifelong genetic exposure, which is different from the short-term effect achieved through adult lipid modification intervention. In addition, the influence of lipid metabolism by genetic factors during embryonic development will also trigger a developmental compensation reaction, which will weaken the estimation of MR-derived effects. Although we have concluded that the level of polytropism is the smallest, it cannot rule out that there may be a compensation mechanism for the real causal relationship that has not been revealed. Therefore, the etiological insights derived from MR should be combined with prospective experiments and clinical intervention research in order to make accurate clinical translation.

More and more studies have linked excessive TG to the pro-inflammatory and estrogen-dominated environment of ectopy endometrial implantation.^[12]^ Hypertriglyceridaemia will increase the output of ultra-LDL in the liver and also enhance the decomposition of fat in the visceral fat bank. The released fatty acids activate NF-κB-driven transcription of IL-6, IL-1β, and TNF-α, which are repeatedly detected in the peritoneal fluid of women with endometriosis.^[13]^ At the same time, the elevation of lipo aromatase will also increase the degree of conversion ofestradiol into androgen in ectopic matrix cells, increase the signal strength of estrogen in ectopic matrix cells, and promote their proliferation and survival.^[14]^ The 3 pathways of lipids, inflammation and hormones formed by this are intertwined, giving our epidemiological findings and molecular pathogenesis a reasonable biological explanation, indicating that TG are a changeable upstream driving factor, not a passive association of disease.

TG plays an important role in various physiological processes. It is related to energy metabolism and storage, to the structural components of cell membranes, and also participates in the regulation of inflammatory response and metabolic homeosis.^[15,16]^ The increase in TG levels will affect the development of endometriosis through different ways. Increased TG levels can lead to an increase in adipose tissue,^[17]^ adipose tissue is an important endocrine organ. The cytokines such as IL-6 and TNF-α secreted by it participate in inflammation and immune response,^[18]^ thus aggravating chronic inflammation in the peritoneal cavity – an important part of the development of endometriosis lesions. The inflammatory environment is not only conducive to the implantation and growth of ectopic endometrial tissue, but also destroys immune monitoring, so that these cells can escape destruction and continue to exist over time.^[19]^ In addition, the link between TG and metabolic syndrome components, namely insulin resistance and visceral obesity, provides a deeper understanding of how lipid imbalance causes endometriosis. Increased TG will destroy lipid metabolism and hormone regulation, mainly increasing the production of estrogen through aromatase activity in fat tissue. Excessive estrogen acts on the extrauterine endometrial tissue and produces proliferation, which in turn causes hormonal imbalance and the development of disease into a vicious circle.^[20,21]^ In addition, the influence of TG on oxidative stress adds a layer of complex factors to this relationship. High TG levels are related to the increase in the production of reactive oxygen. Reactive oxygen can damage cell structure, cause inflammation and enhance the aggressiveness of ectopic endometrial cells.^[22]^ Oxidative stress can accelerate the development of lesions and inhibit local immune responses, resulting in the continuous deterioration and expansion of endometriosis.^[23]^ In summary, these mechanisms show that TG is not only a potential driver of the pathophysiological process of endometriosis.

The increase in TG levels has a certain relationship with endometriosis, which is of great significance in early detection, personalized treatment and comprehensive nursing.^[24]^ Increased TG levels can be found in women with metabolic characteristics related to the high incidence of endometriosis, especially women with previous risk factors. Routine testing of TG levels can provide a basis for lifestyle intervention, that is, reducing TG through diet control and physical exercise. Although reducing TG cannot directly lead to endometriosis or improve symptoms, it can be used as a support for prospective research and intervention research. Future prospective cohort studies and randomized controlled trials should examine whether TG reducers, that is, fibrates or statins, have a preventive effect on women with high risk of high triglyceridemia with endometriosis,^[25]^ and explore whether the combination of these drugs with standard treatment can reduce inflammation and oxidation. Stimulate, and then improve the clinical manifestations of patients.^[26]^ This method is consistent with the principles of precision medicine, in which the treatment is tailor-made according to personal metabolism and hormones. In addition, there is an interaction between TG, metabolic syndrome and endometriosis, highlighting the necessity of comprehensive management of patients. Solving the problem with the components of other metabolic syndromes can provide an overall plan for the management of endometriosis and related diseases (cardiovascular diseases, etc). At this time, public health policies can also play a very crucial role in advocating routine lipid screening, promoting lifestyle improvement and maintaining good lipid levels, so as to reduce the overall impact of endometriosis on the population.^[27]^ Effective clinical and public health interventions for TG management can significantly improve the prevention, treatment and overall quality of life of patients with endometriosis.

This study has an obvious advantage, that is, the use of the NHANES database, which has a wide representation and strong external validity. Secondly, multivariate logical regression and subgroup analysis are used to adjust the key confounding factors and evaluate the heterogeneity of the effect. In addition, Mendel’s randomization analysis application uses genetic variation as a tool variable to provide evidence for causality and reduce the influence of confounding factors. This innovative endometriosis research method provides a new perspective for the study of the disease.

Despite the above advantages, there are also some shortcomings in the study. Although Mendel’s randomization analysis has the ability to infer causality, it still needs longitudinal research to further prove it. However, the data used in the NHANES survey (1999–2006) only represents a short period of time and cannot reflect long-term trends or changes. The diagnosis of endometriosis is based on self-reporting, and there is a certain information bias. Although all covariables have been adjusted, there are still uncontrollable confounding factors, such as lifestyle, eating habits, etc. In addition, the magnetic resonance instruments we use are mainly from individuals of European descent; the frequency and link mode of alleles vary according to different populations, so the size of the effect will also vary. Before these genetic findings are popularized worldwide, it is appropriate to replicate with Asian, African and mixed cohort GWAS.

## 5. Conclusion and outlook

Therefore, our analysis gives convergent evidence of the causal relationship between higher TG and endometriosis, combining the nationally representative NHANES correlation analysis with consistent double-sample MR results with causal effects. Our MR analysis gave consistent genetic evidence that there is a causal relationship with high lifetime TG for the risk of endometriosis. The NHANES analysis also found an observational association in the presence of national representative samples.

It can be seen from the above results that lipid management strategies have the effect of reducing the occurrence of endometriosis. More in-depth research is needed to explain the accurate biological mechanism of the relationship between TG and endometriosis, in terms of inflammation, hormone regulation, etc. Vertical design and larger multicenter cohort studies are needed to confirm and enhance the external validity of the above findings. In addition, the evaluation of the effectiveness of dietary and drug interventions to reduce TG levels and the prevention of endometriosis provides valuable information for the development of new prevention and treatments to alleviate the health problems of women caused by endometriosis.

## Author contributions

**Conceptualization:** Qi Wu, Qi Zhang.

**Data curation:** Qi Zhang, Yifu Tie.

**Writing – original draft:** Qi Wu.

**Writing – review & editing:** Guangyu Cheng.

## Supplementary Material


